# Effects of TGF-β1 Receptor Inhibitor GW788388 on the Epithelial to Mesenchymal Transition of Peritoneal Mesothelial Cells

**DOI:** 10.3390/ijms22094739

**Published:** 2021-04-29

**Authors:** Yunmee Lho, Jun-Young Do, Jung-Yoon Heo, A-Young Kim, Sang-Woon Kim, Seok-Hui Kang

**Affiliations:** 1 Department of Internal Medicine, Smart-Aging Convergence Research Center, College of Medicine, Yeungnam University, Daegu 42415, Korea; ckdwjdgus@naver.com (Y.L.); yuni0325@ynu.ac.kr (J.-Y.H.); 2 Division of Nephrology, Department of Internal Medicine, College of Medicine, Yeungnam University, Daegu 42415, Korea; jydo@med.yu.ac.kr (J.-Y.D.); dkdud0904@naver.com (A.-Y.K.); 3 Division of Gastro-Enterology, Department of Surgery, College of Medicine, Yeungnam University, Daegu 42415, Korea; swkim@med.yu.ac.kr

**Keywords:** peritoneal fibrosis, peritoneal dialysis, Smad activation, transforming growth factor-beta 1

## Abstract

We investigated the effectiveness of the transforming growth factor beta-1 (TGF-β) receptor inhibitor GW788388 on the epithelial to mesenchymal transition (EMT) using human peritoneal mesothelial cells (HPMCs) and examined the effectiveness of GW788388 on the peritoneal membrane using a peritoneal fibrosis mouse model. HPMCs were treated with TGF-β with or without GW788388. Animal experiments were conducted on male C57/BL6 mice. Peritoneal fibrosis was induced by intraperitoneal injection of chlorhexidine gluconate. GW788388 was administered by once-daily oral gavage. The morphological change, cell migration, and invasion resulted from TGF-β treatment, but these changes were attenuated by cotreatment with GW788388. TGF-β-treated HPMCs decreased the level of the epithelial cell marker and increased the levels of the mesenchymal cell markers. Cotreatment with GW788388 reversed these changes. Phosphorylated Smad2 and Smad3 protein levels were stimulated with TGF-β and the change was attenuated by cotreatment with GW788388. For the peritoneal fibrosis mice, thickness and collagen deposition of parietal peritoneum was increased, but this change was attenuated by cotreatment with GW788388. GW788388, an orally available potent TGF-β receptor type 1 inhibitor, effectively attenuated TGF-β-induced EMT in HPMCs. Cotreatment with GW788388 improved peritoneal thickness and fibrosis, and recovered peritoneal membrane function in a peritoneal fibrosis mouse model.

## 1. Introduction

Peritoneal dialysis (PD) is one of the most commonly used dialysis modalities in patients with end-stage renal disease requiring renal replacement therapy. Successful long-term PD requires the maintenance of peritoneal membrane characteristics. Peritoneal fibrosis (PF) is one of the main challenges to maintaining PD [1]. Fibrotic change in the peritoneal membrane can be caused by various conditions, such as exposure to uremia, high-glucose solutions, glucose-degradation products, or peritonitis. Transforming growth factor-beta 1 (TGF-β)-induced epithelial to mesenchymal transition (EMT) is an important pathway underlying the development of PF caused by various conditions [2]. Previous studies investigated the attenuation of TGF-β-induced EMT with various interventions with favorable results [1,3,4]. However, most of these studies did not apply to clinical studies or did not show favorable results in clinical studies. Therefore, additional studies for other interventions or therapeutic drugs to attenuate TGF-β-induced EMT are required.

TGF-β receptors comprise TGF-β receptor type 1 (TβRI) and 2 (TβRII), and through TGF-β binding, TGF-β signaling causes activation of the canonical and non-canonical pathways, which affects EMT in peritoneal mesothelial cells. The binding of TGF-β to the transmembrane TβRII initiates TβRI activation and recruitment. TβRII serine/threonine kinase phosphorylates serine and threonine residues on the transmembrane of TβRI, inducing the phosphorylation of Smad 2 and 3 serine residues [5,6]. The phosphorylated Smad 2 and 3 recruit Smad 4, which is translocated to the nucleus. Consequently, transcription of the TGF-β-targeted gene is initiated, which triggers the transformation of epithelial cells to mesenchymal cells.

GW788388 is a potent TβRI inhibitor and previous studies demonstrated that GW788388 blocked TGF-β1-induced Smad activation and decreased EMT in various tissues/cell lines, including renal tissue, breast cancer cell lines, and cardiac tissue [7,8,9]. Besides, oral GW788388 improved cardiac function and reduced cardiac fibrosis in patients with Chagas heart disease [10]. Considering the importance of TGF-β signaling for EMT of peritoneal mesothelial cells, we expected that GW788388 would block TGF-β signaling, resulting in a decrease in EMT and peritoneal membrane fibrosis. In this study, we investigated the effectiveness of the TGF-β receptor inhibitor GW788388 on the EMT signaling pathway using human peritoneal mesothelial cells (HPMCs) and examined the effectiveness of GW788388 on the peritoneal membrane using a PF mouse model.

## 2. Results

### 2.1. Morphological Change and Cell Migration in TGF-β-Treated HPMCs with or without GW788388

**Figure 1:**
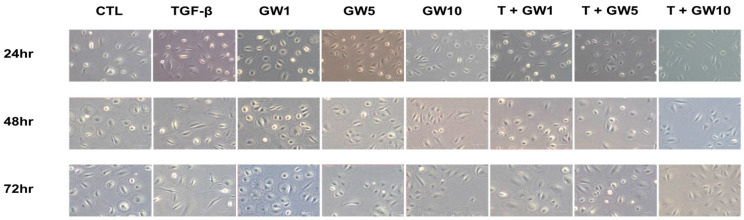
Effects of GW788388 on transforming growth factor-beta 1 (TGF-β)-induced morphological changes (200×). Cobblestone appearance was transformed to spindle shape by TGF-β treatment and the morphological change was attenuated by cotreatment with 1, 5, and 10 μM of GW788388. CTL, control; TGF-β, transforming growth factor-beta 1; GW1, cells exposed to 1 μM of GW788388; GW5, cells exposed to 5 μM of GW788388; GW10, cells exposed to 10 μM of GW788388; T + GW1, cells exposed to TGF-β and 1 μM of GW788388; T + GW5, cells exposed to TGF-β and 5 μM of GW788388; T + GW10, cells exposed to TGF-β and 10 μM of GW788388.

**Figure 2:**
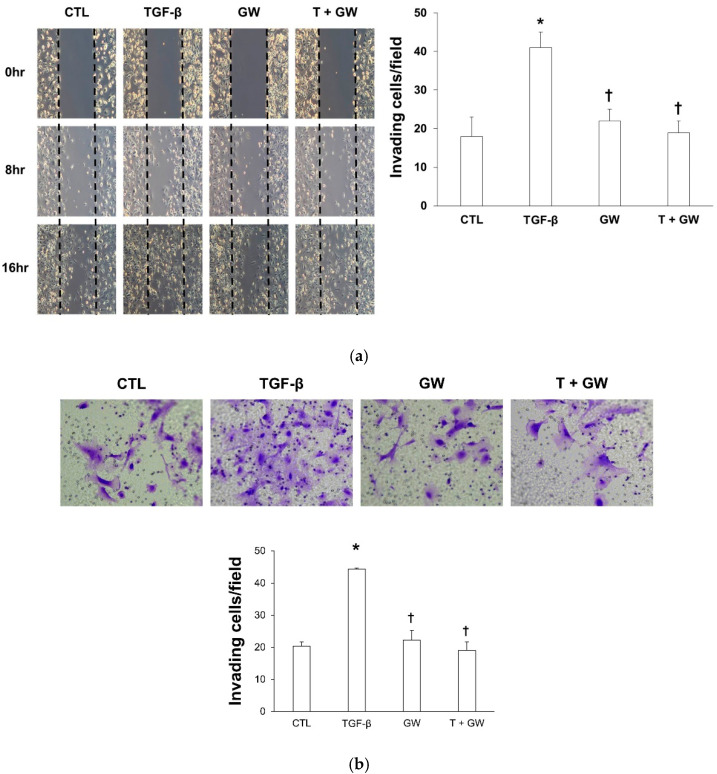
GW788388 inhibits transforming growth factor-beta 1 (TGF-β)-induced cell migration ((**a**), 100×) and invasion ((**b**), 200×). (**a**). Wound healing/migration assay shows that TGF-β-induced wound closure was attenuated by cotreatment with GW788388. (**b**). Matrigel invasion assay shows that TGF-β-induced invasion was attenuated by cotreatment with GW788388. CTL, control; TGF-β, cells exposed to transforming growth factor-beta 1; GW, cells exposed to 1 μM of GW788388; T + GW, cells exposed to TGF-β and 1 μM of GW788388. The invading cells are expressed as mean and standard error (*n* = 3 per group). * *p* < 0.05 compared to cells treated with the control buffer. † *p* < 0.05 compared to cells treated with TGF-β.

The results of the cytotoxicity assay are shown in Figure S1. Cell viability decreased from 100 μM. Figure 1 shows the morphological changes in HPMC after treatment with TGF-β and/or GW788388. The morphological change from cobblestone shape to spindle shape resulted from TGF-β treatment and increased along with an increase in the treatment time. However, this change was attenuated by cotreatment with GW788388. Wound healing/migration assay results showed that cell migration was more prominent in TGF-β-treated HPMCs over time than in normal HPMCs, but GW788388 attenuated cell migration by TGF-β (Figure 2a). Similar trends were observed with cell invasion results (Figure 2b).

### 2.2. Change in EMT Markers in TGF-β-Treated HPMCs with or without GW788388

**Figure 3:**
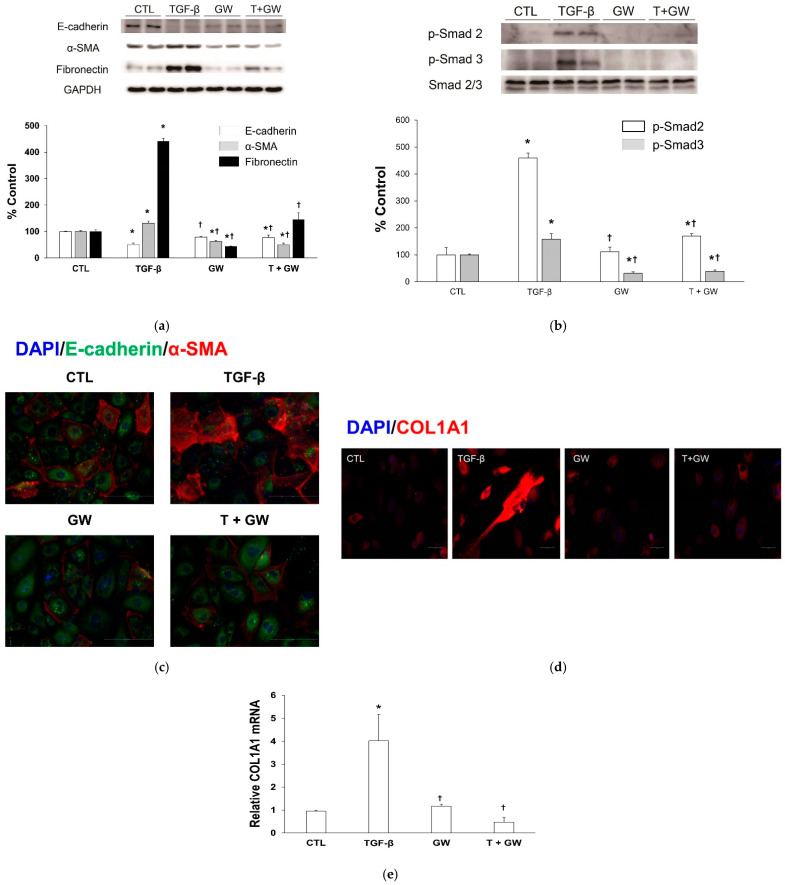
Immunoblotting for epithelial to mesenchymal transition markers (**a**) and the Smad-dependent signaling pathway (**b**), and immunostaining of E-cadherin (green) and α-SMA (red) with nuclear counterstaining (DAPI; blue) in human peritoneal mesothelial cells ((**c**), 400×). Immunostaining of COL1A1 ((**d**), 400×) and the expression of *COL1A1* mRNA in human peritoneal mesothelial cells (**e**). CTL, control; TGF-β, cells exposed to transforming growth factor-beta 1; GW, cells exposed to 1 μM of GW788388; T + GW, cells exposed to TGF-β and 1 μM of GW788388; α-SMA, α-smooth muscle actin; p-Smad2, phospho-Smad2; p-Smad3, phospho-Smad3; COL1A1, collagen type 1, alpha 1. The percentage for the control is expressed as mean and standard error (*n* = 3 per group). * *p* < 0.05 compared to cells treated with the control buffer. † *p* < 0.05 compared to cells treated with TGF-β.

TGF-β-treated HPMCs decreased the level of the epithelial cell marker E-cadherin and increased the levels of the mesenchymal cell markers α-smooth muscle actin (α-SMA) and fibronectin (Figure 3a). Cotreatment with GW788388 reversed these changes. Phosphorylated Smad2 and Smad3 protein levels were stimulated with TGF-β and the change was attenuated by cotreatment with GW788388 (Figure 3b). Immunostaining of DAPI, E-cadherin, and α-SMA revealed similar trends in epithelial or mesenchymal markers to those from western blotting (Figure 3c). Immunostaining revealed that TGF-β-treated HPMCs increased the expression of collagen type 1, alpha 1 (COL1A1), and cotreatment with GW788388 reversed this change (Figure 3d). The expression levels of *COL1A1* mRNA showed similar trends to those observed following immunostaining (Figure 3e).

### 2.3. Effects of GW788388 in a Mouse Model of PF

Body weight from D0 to D14 was similar among the three groups, but was lower in the PF and PF + GW groups than in the CTL group at D21 (Figure S2). However, no significant difference in food intake occurred among the three groups (Table 1).

The peritoneal equilibrium test showed a decreased D/D0 glucose ratio in the PF group compared with the CTL group, but the ratio was recovered in the PF + GW group compared with the PF group (Figure S3).

**Figure 4:**
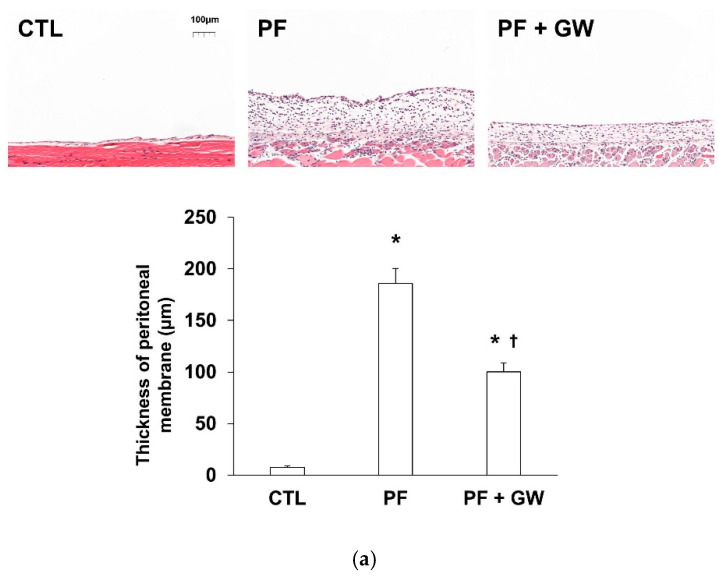

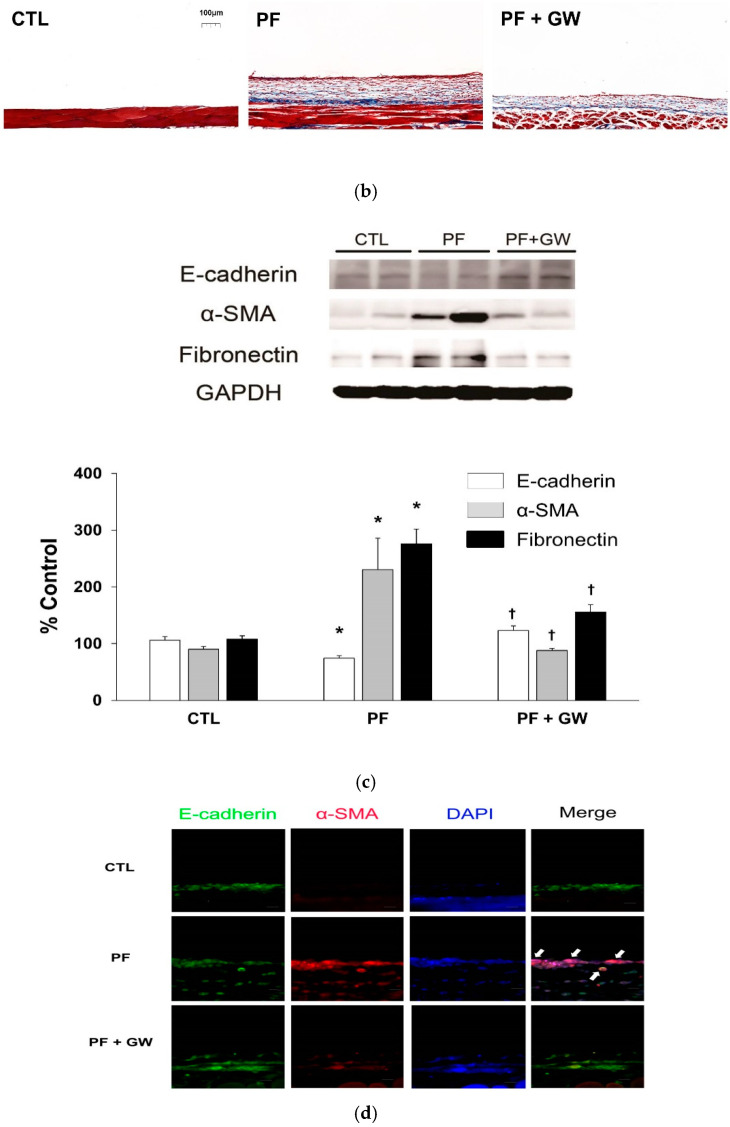
Morphological changes, immunoblotting, and immunostaining results in the peritoneum. (**a**) Hematoxylin and eosin staining (magnification 100×). (**b**) Masson’s trichrome staining (magnification 100×). (**c**) Immunoblotting for epithelial to mesenchymal markers. ((**d**), 400×) Immunostaining for E-cadherin (green) and α-SMA (red) with nuclear counterstaining (DAPI; blue) in the peritoneum. Dual-stained E-cadherin and α-SMA-positive cells are indicated by arrows. CTL, control; PF, mice injected 0.1% chlorhexidine gluconate intraperitoneally; PF + GW, mice injected 0.1% chlorhexidine gluconate intraperitoneally and administered GW788388 orally by gavage; α-SMA, α-smooth muscle actin. The thickness of the peritoneal membrane is expressed as mean and standard error (*n* = 5 for CTL group, *n* = 7 for PF group, *n* = 9 for PF + GW group). * *p* < 0.05) compared to the CTL group. † *p* < 0.05 compared to the PF group.

For the PF group, thickness and collagen deposition of the parietal peritoneum increased, but this change was attenuated by cotreatment with GW788388 (Figure 4A,B). Besides, E-cadherin level was decreased and α-SMA and fibronectin levels were increased compared with the CTL group (Figure 4C); however, these changes were reversed by GW788388 cotreatment. Immunostaining for DAPI, E-cadherin, and α-SMA also showed similar trends to those of western blotting. The PF group had dual positive cells for E-cadherin and α-SMA, but the number of cells decreased with cotreatement with GW788388 (Figure 4D). TGF-β-specific staining revealed an increase in the expression of TGF-β in the PF group, which was decreased following cotreatment with GW788388 (Figure S4). Histological staining for collagen I or fibronectin staining showed an increase in the staining of these factors within the PF group, but the change was attenuated by the cotreatment of GW788388 (Figure S5).

## 3. Discussion

The peritoneal membrane becomes thicker and more fibrous with increasing PD duration. When the thickness and fibrosis of the peritoneal membrane exceeds a threshold, ultrafiltration failure develops. Consequently, ultrafiltration failure is associated with insufficient volume control in patients with PD. A previous study showed that the prevalence of ultrafiltration failure in patients with PD was 18%, and most of these patients are transferred to hemodialysis [11]. Furthermore, severe diseases such as encapsulating peritoneal sclerosis can develop. The frequency of encapsulating peritoneal sclerosis was relatively low, but the prevalence steeply increased as the PD duration increased [12,13]. The mortality of patients with encapsulating peritoneal sclerosis ranges between 26% and 58% [13,14,15]. These pathological changes were caused by bioincompatible dialysate, uremic condition, or peritonitis [2,10,11]. Treatment options include PD discontinuation, nutritional support, use of immunosuppressants, such as glucocorticosteroid, rapamycin, azathioprine, tamoxifen, and surgical treatment [8]. Previous studies have shown a favorable effect of experimental approaches such as stem-cell therapy, colchicine, N-acetylcysteine, pentoxifylline, or rosiglitazone on PF [16,17,18,19,20]. However, most of the treatment options lack sufficient evidence and conservative treatment, such as discontinuation of PD or nutritional support, remains the main option for PF.

GW788388 is a potent TβRI-specific inhibitor [21]. Although previous studies have investigated the favorable effects of HPMCs on EMT using some treatments, such as paricalcitol, tranilast, or metformin, a specific targeted therapy such as GW788388 may have greater potency and less opposing effect than these [1,3,4]. Gellibert et al. first discovered GW788388 as a potent, selective, orally active TβRI inhibitor [21]. GW788388 was derived from phenylpyrazole and showed proper cellular potency and better pharmacokinetics in an in vivo model compared to other phenylpyrazole derivatives. They evaluated the effect of GW788388 using dimethylnitrosamine-induced liver disease and puromycin aminonucleoside nephropathy, and attenuated the expression of collagen [21]. A previous study showed that GW788388 attenuated TGF-β-induced EMT responses of renal epithelial cells and decreased glomerulopathy and renal fibrosis in db/db mice [7]. Derangeon et al. investigated the long-term effects of GW788388 on myocardial fibrosis and showed that myocardial fibrosis via sodium channel loss was attenuated by a 15-week GW788388 treatment [6]. Previous studies in relation to chronic Chagas heart disease or myocardial infarction showed similar results [10,22]. The anti-fibrotic effect of GW788388 was also evaluated in fibrosis models using acetaminophen-induced liver injury and submandibular gland ligation [23,24].

GW788388 was first identified as a selective inhibitor of TβRI (activin-like receptor kinase [ALK] 5) expression. Petersen et al. showed that in addition to TβRI, GW788388 could inhibit ALK4, ALK7, and TβRII expression, with no inhibitory effect on the levels of BMP receptor [5]. These findings suggest that GW788388 affects TGF-β-specific inhibition regardless of BMP signaling. Our study did not evaluate the effect of GW788388 on the expression of TGF-β receptor subtypes. Although the inhibitory potency against TβRI expression may be stronger than that against TβRII expression, GW788388 possibly inhibits the levels of both TβRI and TβRII. Moreover, the final signaling pathway mediated by GW788388 does not differ based on the inhibitory strength toward TβRI and TβRII expression. Furthermore, TGF-β-specific inhibition of GW788388 may apply to some pathological conditions. Interestingly, Levolger evaluated the effect of GW788388 on a cancer cachexia model and showed favorable results [25]. Myostatin or activin, a negative regulator of muscle growth inhibitors, binds to the activin receptor type II receptor, which recruits TβRI, ALK4, or ALK7 [26]. These initiate a signaling pathway via the phosphorylation of Smad 2 and 3, which results in muscle wasting. These studies reveal that GW788388 can inhibit muscle wasting in cancer by inhibiting the expression of TβRI, ALK4, or ALK7.

A previous study focused on the effect of GW788388 on EMT in nonmalignant, premalignant, and malignant mammary cells and showed that GW788388 effectively attenuates the change in TGF-β-induced EMT [9]. Although previous studies showed the effect of GW788388 in some cells or organs, to our best knowledge, very little data exists on the effect of GW788388 on TGF-β-induced EMT in peritoneal mesothelial cells. First, we harvested HPMCs from the human omentum and EMT was evaluated based on changes in morphology and epithelial or mesenchymal markers. GW788388 attenuated morphological changes induced by TGF-β, and it reversed the TGF-β-mediated changes in epithelial or mesenchymal markers. These changes were also confirmed by wound healing/migration and invasion tests. Furthermore, we used a chlorhexidine gluconate (CG)-induced PF model and showed similar results in vitro.

Our study has some limitations. First, in our experiments, the EMT in HPMCs was induced by TGF-β and EMT in the in vivo model was induced by an intraperitoneal CG injection. The exact model of EMT induction would be closer to EMT induced using peritoneal dialysate than with TGF-β. Besides, CG-induced PF can be strong compared to peritoneal dialysate-induced PF. Strong injury in the peritoneum may underestimate the real effect of GW788388. Although we did not evaluate the association between PF and TGF-β expression in mouse peritoneal mesothelial cells, TGF-β staining results in our animal model showing similar trends to those observed using mouse peritoneal mesothelial cells. Finally, human data for GW788388 are lacking owing to insufficient information regarding GW788388 safety. These are important hurdles to be overcome for application in clinical settings. Therefore, further preclinical studies regarding the safety of GW788388 should be considered to overcome this limitation.

In conclusion, GW788388, an orally available potent TβRI inhibitor, effectively attenuated TGF-β-induced EMT in HPMCs. Cotreatment with GW788388 improved peritoneal thickness and fibrosis, and recovered peritoneal membrane function in a PF mouse model. This knowledge may be beneficial for further pre-clinical study considerations regarding safety, prevention, and treatment of PF.

## 4. Materials and Methods

### 4.1. In Vitro Study

#### 4.1.1. HPMC Culture and Treatment Conditions

This study was approved by the Institutional Review Board of Yeungnam University Medical Center (approval no, 2019-09-023; date, 15-11-2019). At our center, gastrectomy was considered for all biopsy-proven patients with stomach cancer, except in patients with inoperable or endoscopically resectable lesions. Surgery was performed using partial or total gastrectomy with total or partial omentectomy. For cases with omentectomy, the lymph nodes were resected and transferred for pathological analysis. The remnant omentum was inspected by the surgeon to determine whether definite local invasion or metastasis had occurred. If a definite local invasion was not detected, the remnant omentum was discarded. Prior to surgery, informed consent was obtained after explaining the purpose and methods of our study (treatment/pathophysiology of PF and usage of mesothelial cells from the discarded omentum). Omentum without definite local invasion or metastasis was included in our study. HPMCs were isolated as described previously [3,4]. In vitro experiments were performed on cells after 1 to 2 passages. HPMCs were incubated with serum-free M199 medium (31100, Gibco, Thermo Scientific, Waltham, MA, USA) for 24 h. Subsequently, HPMCs were cultured in M199 medium supplemented with l-glutamine (Gibco, 20 μM/mL), penicillin-streptomycin (Gibco, 150 units or 150 μg/mL), hydrocortisone (Sigma, St. Louis, MO, USA, 0.4 μg/mL), apotransferrin (Sigma, 5 μg/mL), insulin (Sigma, 5 μg/mL), and 10% fetal bovine serum (FBS) (Gibco). Cell cultures were maintained in a 100-mm culture dish (Nunc, Roechester, NY, USA) in a 95% air and 5% CO_2_ humidified atmosphere incubator (311 Forma Direct Heat CO_2_ incubator, Thermo Scientific, Waltham, MA, USA) at 37 °C.

#### 4.1.2. Cytotoxicity Assay

The cytotoxicity of GW788388 (SML0116, Sigma-Aldrich, St. Louis, MO, USA) was evaluated with the Cell-Counting Kit-8 (CCK-8) (CK04, Dojindo Laboratories, Tokyo, Japan). HPMCs (10,000 cells) were cultured in a 96-well plate (0494, Corning, Bedford, MA, USA) for 24 h and then treated with GW788388 (0, 0.01, 0.1, 1, 10, 100, and 1000 μM) for 24–72 h. The CCK-8 solution was added to each well, and optical density at 450 nm was measured between 1 and 4 h. The wells were read using a 96-well plate reader (Synergy HTX, BioTek Instruments Inc., Winooski, VT, USA). 

#### 4.1.3. Cell morphology and Wound Healing and Invasion Tests

HPMCs grown in a culture dish were incubated in an M199 culture medium for 24 h for cell cycle synchronization. After this period, the growth media were substituted with a serum-free M199 medium supplemented with 1% FBS and 2.0 ng/mL TGF-β1 (R&D Systems, Minneapolis, MN, USA) with or without GW788388 (1, 5, or 10 μM) for 24–72 h. Cell morphology was analyzed under an inverted phase-contrast microscope (200×) (DMi8, Leica, Wetzlar, Germany).

We adopted the wound healing/migration assay to detect cell migration. HPMCs were seeded at a density of 10,000 cells/well in a culture-insert-2 well (81176, Ibidi GmbH, Martinsried, Germany). After allowing the cells to attach overnight, we removed the culture-insert and washed the cells with phosphate-buffered saline (PBS) (PR2007-100-00, Biosesang, Seongnam, Korea) to remove non-adherent cells. We then provided a fresh medium containing serum-free M199 with 1% FBS and TGF-β (2.0 ng/mL) with or without GW788388 (1 μM) and photographed the plate at 0, 8, and 16 h to capture the two fields at each time point on each plate. The number of cells that migrated to the wound space was manually counted (in three fields per well) under a light microscope (DMi8, Leica, Wetzlar, Germany) at 200× magnification [27].

The cell invasion assay was evaluated using transwell inserts containing an 8-µm pore size polycarbonate membrane (3422, Corning, Bedford, MA, USA), according to the protocol described previously, but with slight modification [28]. Briefly, the inner surface of the membrane was coated with 20 µL of Matrigel (0.5 mg/mL, 354234, Corning, Bedford, MA, USA) and incubated overnight at 37 °C to solidify the Matrigel, and the outer surface was coated with 30 µL of type I collagen (0.5 mg/mL, 354236, Corning, Bedford, MA, USA) and incubated for 3 h at 37 °C. After drying, 100-µL serum-free M199 medium with 2% FBS and 700-µL serum-free M199 medium containing 10% FBS were added to the upper and lower chambers of the transwell system. After conventional digestion, cells in each group were resuspended in a serum-free M199 medium at a density of 100,000 cells/flask. A total of 200-µL cell suspension was added to the upper chamber of the transwell system on the top of the Matrigel coating. After incubation at 37 °C for 16 h, the invaded cells were fixed with 4% paraformaldehyde (pc2031-100-00, Biosesang, Seongnam, Korea) for 10 min, methanol for 5 min, and 0.5% crystal violet solution (V5265, Sigma-Aldrich, St. Louis, MO, USA) prepared with methanol for 1 h, and the stained cells were counted using a microscope (×200) (DMi8, Leixca, Wetzlar, Germany).

#### 4.1.4. Western Blotting

Primary antibodies against E-cadherin (610181, BD biosciences, Franklin Lakes, NJ, USA) and α-SMA (A2547, Sigma-Aldrich, St. Louis, MO, USA) (1:1000) and the corresponding horseradish peroxidase (HRP)-conjugated secondary anti-mouse IgG (sc-516102, Santa Cruz Biotechnology, Dallas, TX, USA) (1:2000); primary antibodies against fibronectin (ab268020, Abcam, Cambridge, MA, USA), GAPDH (#2118, Cell signaling Technology, Boston, MA, USA), phospho-Smad2 (#3108, Cell signaling Technology, Boston, MA, USA), phospho-Smad3 (#9520, Cell signaling Technology, Boston, MA, USA), and Smad2/3 (#3102, Cell signaling Technology, Boston, MA, USA) (1:1000) and the corresponding HRP-conjugated secondary anti-rabbit IgG (A16096, Invitrogen, Thermo Fisher Scientific, Waltham, MA, USA) (1:2000) was purchased and diluted at indicated concentrations.

Harvested HPMCs and tissues were subjected to 10% SDS-PAGE on appropriate resolving gels and immunoblotted. Briefly, tissues and cells were lysed in ice-cold RIPA buffer (25 mM Tris-HCl (pH 7.6), 150 mM NaCl, 1% NP-40, 1% sodium deoxycholate, 0.1% SDS; (89901, Thermo Scientific, Waltham, MA, USA)) containing 1% protease inhibitor single-use cocktail solution (100×) ((1 mM AEBSF, 800 nM aprotinin, 50 μM, bestatin, 15 μM E64, 20 μM leupeptin, 10 μM pepstatin A, and 5 mM EDTA (78430, Thermo Fisher Scientific, Waltham, MA, USA)). The lysates were centrifuged at 7500 rpm for 30 min at 4 °C and the supernatant was collected. Proteins were separated by 10% SDS-PAGE and transferred to a nitrocellulose membrane (10600023, Amersham, GE Healthcare Life Science, Chicago, IL, USA). The membrane was blocked with 5% skim milk (MB-S1667, MB cell, Korea) in TBS-T (247 mM Tris, 1.37 M sodium chloride, 27 mM potassium chloride, and 0.5% Tween 20 (pH 7.4), (TR2007-100-74, Biosesang, Seongnam, Korea)) before incubation overnight at 4 °C with the primary antibodies. The membrane was then washed with TBS-T and incubated with the HRP-conjugated secondary antibodies. Protein bands were detected using enhanced chemiluminescent reagents (34095, Thermo Fisher Scientific, Waltham, MA, USA). Membranes were detected using LAS-3000 (Fujifilm, Tokyo, Japan) and the areas were quantified using ImageJ software [29].

#### 4.1.5. Immunofluorescence

HPMCs (20,000 cells) were grown in an 8-well chamber slide (154534, Thermo Fisher Scientific, Waltham, MA, USA) for 24 h for cell-cycle synchronization. The growth media were then substituted for serum-free M199 medium supplemented with 1% FBS and TGF-β (2.0 ng/mL) with or without GW788388 (1 μM) for 24 h. For immunofluorescence staining, cells were washed in 1% bovine serum albumin (BSA) (160069, MP biomedicals, Illkirch, Solon, OH, USA) dissolved in PBS and fixed in 4% paraformaldehyde (10 min at 4 °C). Then, cells were permeabilized with 0.1% Triton X-100 in PBS (15 min at 4 °C) and washed again with 1% BSA in PBS. After incubation overnight at 4 °C with primary antibodies against E-cadherin (610181, dilution 1:100, BD biosciences, Franklin Lakes, NJ, USA), α-SMA (#19245, dilution 1:200, Cell signaling Technology, Boston, MA, USA), COL1A1 (GTX20292, dilution 1:1000, GeneTex, Irvine, CA, USA), and TGF-β (ab215715, dilution 1:200, Abcam, Cambridge, MA, USA), the cells were treated with 1% BSA in PBS for 1 h and subsequently washed again with PBS. Sections were incubated for 1 h with fluorescein-conjugated secondary antibodies, Alexa Fluor 488-conjugated goat anti-mouse IgG (A11001, Invitrogen, Thermo Fisher Scientific, Waltham, MA, USA), and Alexa Fluor 568 goat anti-rabbit IgG (A11012, Invitrogen, Thermo Fisher Scientific, Waltham, MA, USA). A slide chamber was mounted using a mounting medium containing DAPI (H-1200, Vector Laboratories, Burlingame, CA, USA) in the dark at room temperature. The nucleus was counterstained with DAPI, and the stained slide chamber was examined under a microscope (400×) (DMi8, Leica, Wetzlar, Germany).

#### 4.1.6. Real-Time Reverse Transcriptase Polymerase Chain Reaction (Real-Time RT-PCR) Analysis

Real-time RT-PCR was performed using the iQ™ SYBR^®^ Green Supermix (1708880AP, Bio Rad, Singapore, Singapore). The RT-PCR reaction was performed with 10 μL of the iQ™ SYBR^®^ Green Supermix, 1 μL of 10 pmole/μL forward and reverse primers, 6 μL of water, and 2 μL of template cDNA for a final volume of 20 μL. The primer sequences were as follows: COL1A1—forward, 5′-GCCTCAAGGTATTGCTGGAC-3′, reverse, 5′-ACCTTGT TTGCCAGGTTCAC-3′; β-actin—forward, 5′-ATCGTGCGTGACATTAAGGA-3′, reverse 5′-ATTGCCAATGGTGATGACCTG-3′. The relative mRNA expression levels of the target genes in each sample were calculated using the comparative CT method. The CT value is the cycle number at which the fluorescence signal is greater than a defined threshold. The relative expression of each gene was normalized against β-actin. The samples were assayed on a CFX Connect real-time System (CFX connect Optics Module, Bio rad, Singapore, Singapore) instrument.

### 4.2. In Vivo Study

#### 4.2.1. Animal Experiments

All experiments were conducted on male C57/BL6 mice (10 weeks, 20–25 g) (Samtako Biokorea, Seoul, Korea). Mice were group-housed under a 12:12 h light:dark cycle at 24 ± 1 °C. Mice had unrestricted access to standard tap water, and they were allowed to acclimate to the environment for at least 7 days. All animal procedures were approved by the Institutional Review Board of Yeungnam University College of Medicine (YUMC-2020-033) and were in accordance with the Guide for the Care and Use of Laboratory Animals.

PF was induced in mice following a protocol described previously, but with a slight modification [30]. Mice were classified into 3 groups: (i) CTL (*n* = 5), in which 15% ethanol dissolved in PBS was intraperitoneally injected into the mice every other day and oral gavage using 30% PEG400 (25322-68-3, Sigma Aldrich, St. Louis, MO, USA), 0.5% Tween 80 (P1754, Sigma Aldrich, St. Louis, MO, USA), and propylene glycol (P4347, Sigma Aldrich, St. Louis, MO, USA) in water (0.2 mL/body) was performed daily; (ii) PF (*n* = 7), in which 0.1% CG and 15% ethanol dissolved in PBS were intraperitoneally injected into mice every other day and oral gavage using 30% PEG400, 0.5% Tween 80, and propylene glycol in water (0.2 mL/body) was performed daily; (iii) PF + GW (*n* = 9), in which 0.1% CG and 15% ethanol dissolved in PBS were intraperitoneally injected into mice every other day and oral gavage using GW788388 (5 mg/kg/day) with 30% PEG400, 0.5% Tween 80, and propylene glycol in water (0.2 mL/body) was performed daily [3]. To avoid any artifacts arising from peritoneal damage due to repeated injections, 0.1% CG was injected at the lower part of the peritoneum, and the upper portion of the parietal peritoneum was used for the analyses. The mice were euthanized at 21 days. All mice were anesthetized via intraperitoneal injection of a tiletamine (125 μg/g), zolazepam (125 μg/g) (Vibac, Seoul, Korea), and xylazine (0.78 μg/g) (260, Bayer-Korea, Seoul, Korea) combination.

#### 4.2.2. Peritoneal Membrane Function Test

Mice in each group underwent a peritoneal equilibrium test on the last day before euthanasia. Mice were injected with 2 mL of 4.25% glucose solution containing the dialysate (Physioneal^®^, Baxter Healthcare, Singapore, Republic of Singapore) for 2 h and then euthanized to collect the dialysate. Glucose levels in the dialysate were determined according to the manufacturer’s instructions (Seoul Clinical Laboratories, Yongin, Korea). Functional alteration of peritoneal membranes was evaluated by the D/D0 glucose level using the dialysate glucose level at 2 h after dialysate infusion per the level at 0 h [31]. 

#### 4.2.3. Morphometric and Immunohistochemical Analyses of the Peritoneum

The parietal peritoneum of the abdominal wall was fixed with 4% paraformaldehyde, embedded in paraffin, and then cut into 4-μm thick sections. The thickness of the parietal peritoneum, including the mesothelium and submesothelial tissue, was measured in tissue sections under a microscope (DMi8, Leica, Wetzlar, Germany) after staining with hematoxylin and eosin. The slide scans were performed using a system panoramic digital slide scanner (Panoramic scan2, 3Dhistech Ltd., Budapest, Hungary) and thickness was measured using the Caseviewer software (3Dhistech Ltd., Budapest, Hungary). To enhance the detection of fibrosis, the sections were stained with Masson’s trichrome stain (Trichrome stain kit, TRM-2, ScyTek, Logan, UT, USA). 

For immunohistochemistry, tissues were fixed with 4% paraformaldehyde (pH 7.4), embedded in paraffin, and cut into 4-μm sections using a microtome. Tissue sections were rehydrated using xylene and a series of graded ethanol. After washing the sections with water for 5 min, the sections were permeabilized using 3% H_2_O_2_ (1145, Duksan, Ansan, Korea) in methanol for 15 min, then washed again twice with water for 5 min. For antigen unmasking, sections were immersed in citrate buffer (150 mM sodium citric-acid, pH 6.0), boiled for 10 min, cooled at room temperature for 20 min, washed with water, and blocked with 5% normal goat serum-blocking solution (NGS) (S-1000-20, Vector Laboratories, Burlingame, CA, USA) in PBS for 30 min. The sectioned tissues were incubated with anti-collagen I (GTX 20292, dilution 1:200, GeneTex, Irvine, CA, USA), anti-fibronectin (ab268080, dilution 1:200, Abcam, Cambridge, MA, USA), and anti-TGF-β antibodies (ab215715, dilution 1: 200, Abcam, Cambridge, MA, USA) in 5% NGS overnight at 4 °C. The sections were then washed with PBS followed by incubation with goat anti-rabbit antibody (A16096, dilution 1:200, Invitrogen, Thermo Fisher Scientific, Waltham, MA, USA) in 5% NGS at room temperature for 1 h. Subsequently, they were washed with PBS for 10 min, stained with diaminobenzidine (as a substrate for the enzyme complex) according to the manufacturer’s instructions (DAB Substrate kit for peroxidase, SK-4105, Vector Laboratories, Burlingame, CA, USA), and counterstained with hematoxylin (S3309, DAKO, Carpinteria, CA, USA). Sections were then dehydrated using a series of graded ethanol, cleared in xylene, and covered with coverslips using the mounting medium solution (3801120, Leica, Wetzlar, Germany). For immunofluorescence microscopy, 15-μm tissue sections were incubated; the process was the same as for the in vitro samples.

### 4.3. Statistical Analysis

IBM SPSS Statistics (version 25.0, IBM Corp., Armonk, NY, USA) was used to analyze the data. Data are expressed as the means and standard errors. Groups were compared by the Kruskal–Wallis or Mann–Whitney rank-sum test. Differences between the two time points were compared by the Wilcoxon signed-rank test. A *p* < 0.05 was considered significant. 

## Figures and Tables

**Table 1:** Chow intake during the experiment.

	CTL	PF	PF + GW	*p*-Value ^1^
D1 to D7 (g/day)	3.0 ± 0.4	2.8 ± 0.2	3.2 ± 0.3	0.129
D8 to D14 (g/day)	3.1 ± 0.2	2.8 ± 0.2	3.4 ± 0.2	0.071
D15 to D21 (g/day)	3.3 ± 0.0	2.8 ± 0.2	3.1 ± 0.3	0.252

^1^ Comparison was performed using the Kruskal–Wallis test. Data are expressed as mean and standard error (*n* = 5 for CTL group, *n* = 7 for PF group, *n* = 9 for PF + GW group). CTL, control; PF, mice injected 0.1% chlorhexidine gluconate intraperitoneally; PF + GW, mice injected 0.1% chlorhexidine gluconate intraperitoneally and administered GW788388 orally by gavage; D1 to D7, mean chow intake between days 1 and 7; D8 to D14, mean chow intake between days 8 and 15; D15 to D21, mean chow intake between days 16 and day 21.

## Data Availability

Data are contained within the article and supplementary material.

## Supplementary Materials

The following are available online at https://www.mdpi.com/article/10.3390/ijms22094739/s1, Figure S1. Cytotoxic effects of GW788388 on human peritoneal mesothelial cells. GW788388 (0, 0.01, 0.1, 1, 10, 100, and 1000 μM) was treated for 24, 48, or 72 h. GW788388 was not significantly toxic up to a concentration of 10 μM. The cell viabilities are expressed as mean and standard error (*n* = 3 per group). * *p* < 0.05 compared to cells treated with the control buffer. Figure S2. Body weight during experiment. CTL, control; PF, mice injected with 0.1% chlorhexidine gluconate intraperitoneally; PF + GW, mice injected 0.1% chlorhexidine gluconate and administered GW788388 orally by gavage. The data are expressed as mean and standard error (*n* = 5 for CTL group, *n* = 7 for PF group, *n* = 9 for PF + GW group). * *p* < 0.05 compared to the CTL group. Figure S3. Peritoneal equilibrium test. CTL, control; PF, mice injected 0.1% chlorhexidine gluconate intraperitoneally; PF + GW, mice injected 0.1% chlorhexidine gluconate and administered GW788388 orally by gavage; D/D0 glucose, the ratio of dialysate glucose level at 2 h after dialysate infusion per the level at 0 h. The data are expressed as mean and standard error (*n* = 5 for CTL group, *n* = 7 for PF group, *n* = 9 for PF + GW group). * *p* < 0.05 compared to the CTL group. ^#^ *p* < 0.05 compared to the PF group. Figure S4. TGF-β expression changes in the peritoneum. Immunostaining (a, 400×) and immunohistochemical staining (b, 200×) of TGF-β. CTL, control; PF, mice injected with 0.1% chlorhexidine gluconate intraperitoneally; PF + GW, mice injected with 0.1% chlorhexidine gluconate intraperitoneally, and GW788388 was administered orally by gavage; TGF-β, transforming growth factor beta-1. Figure S5. Morphological changes in the peritoneum. The peritoneum was stained with collagen I (a) and fibronectin (b) (200×). CTL, control; PF, mice injected 0.1% chlorhexidine gluconate intraperitoneally; PF + GW, mice injected 0.1% chlorhexidine gluconate intraperitoneally and administered GW788388 orally by gavage.
