# Supplementary figures and images for: Effects of TGF-β1 Receptor Inhibitor GW788388 on the Epithelial to Mesenchymal Transition of Peritoneal Mesothelial Cells
Source: Int J Mol Sci. 2021 Apr 29;22(9):4739. doi: 10.3390/ijms22094739 (PMC8124410; doi:10.3390/ijms22094739)

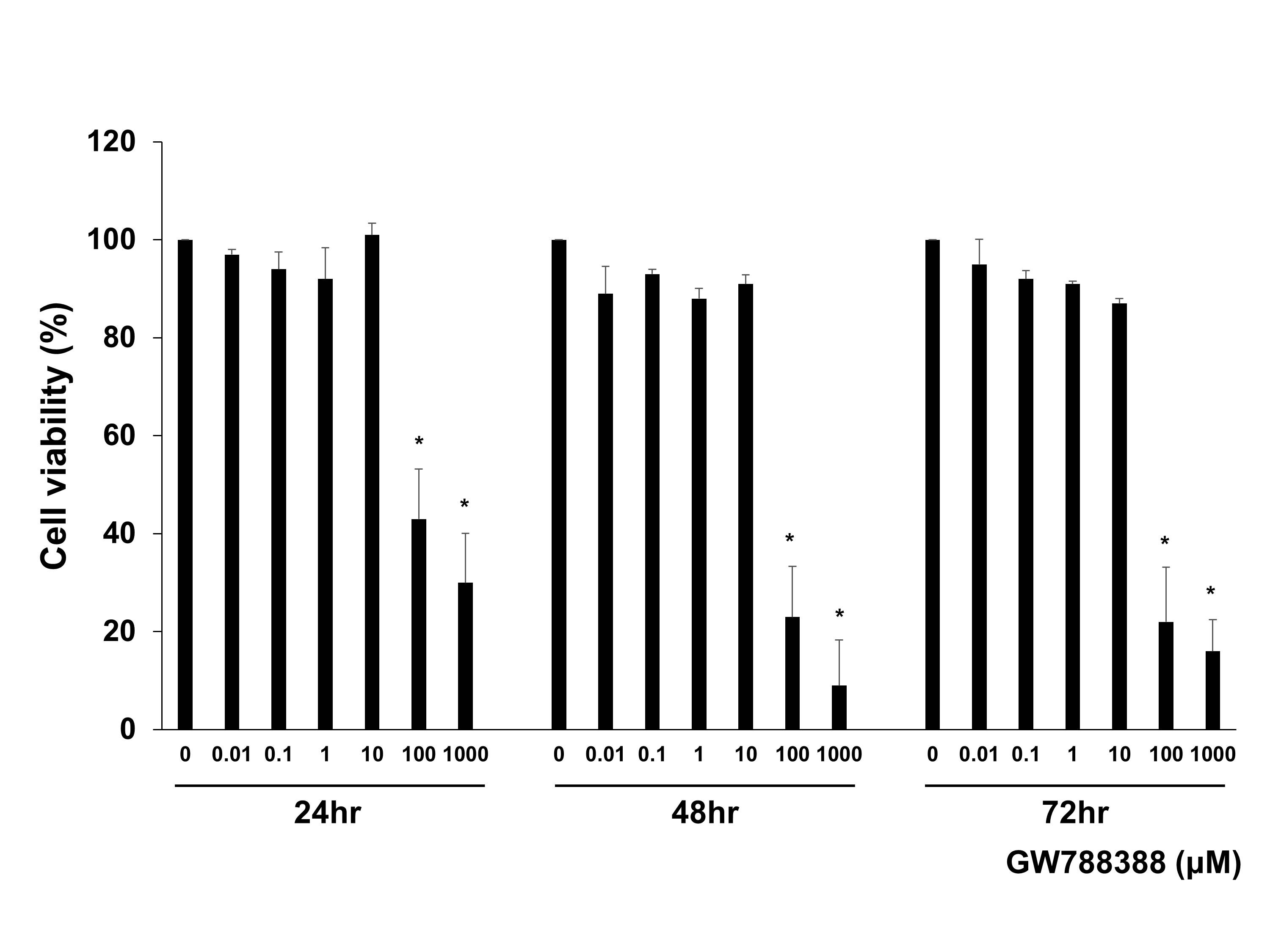

Supplement: Supplementary file 1 [file ijms-22-04739-s001.zip › Figure S1.jpg]

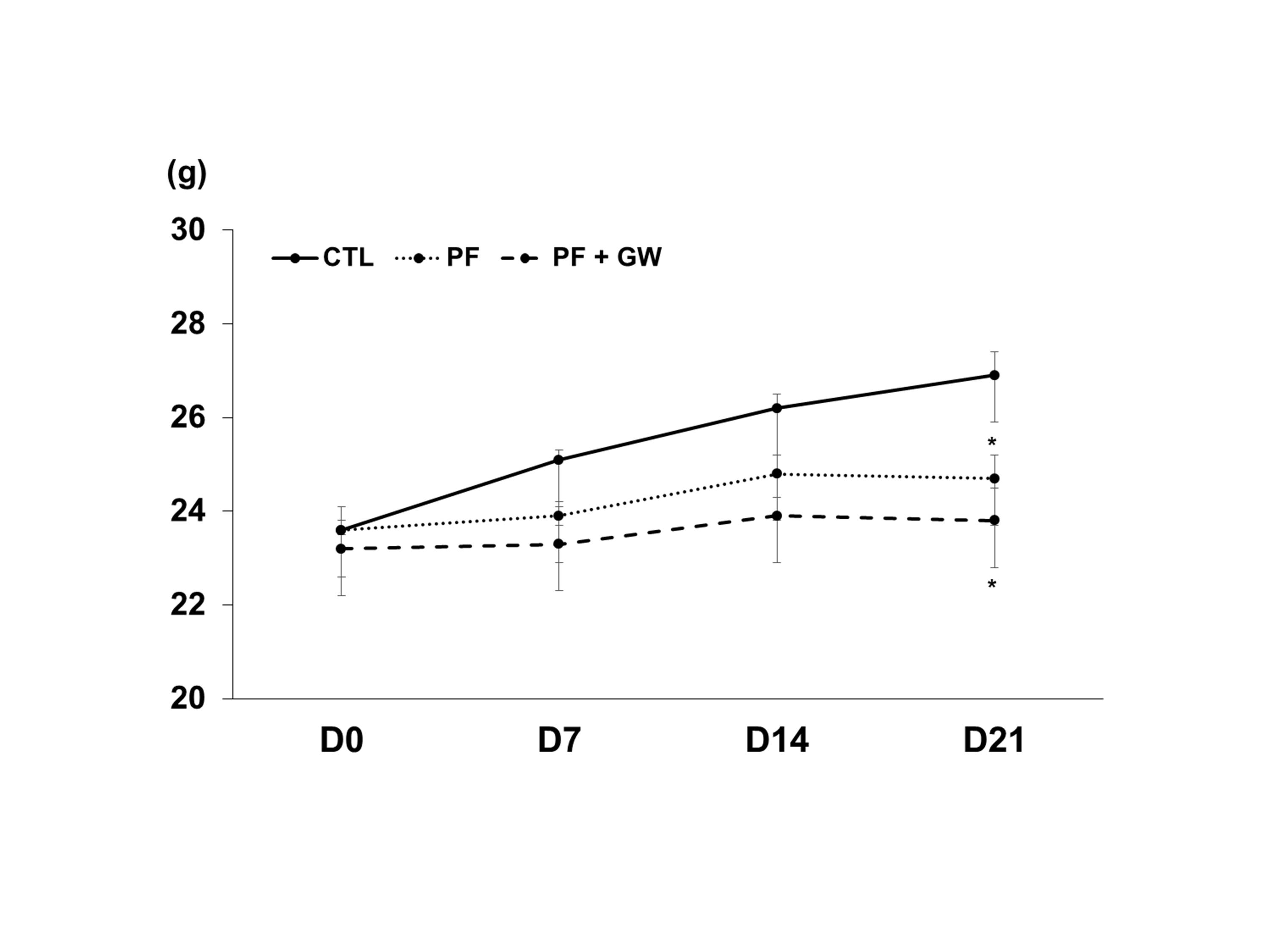

Supplement: Supplementary file 1 [file ijms-22-04739-s001.zip › Figure S2.jpg]

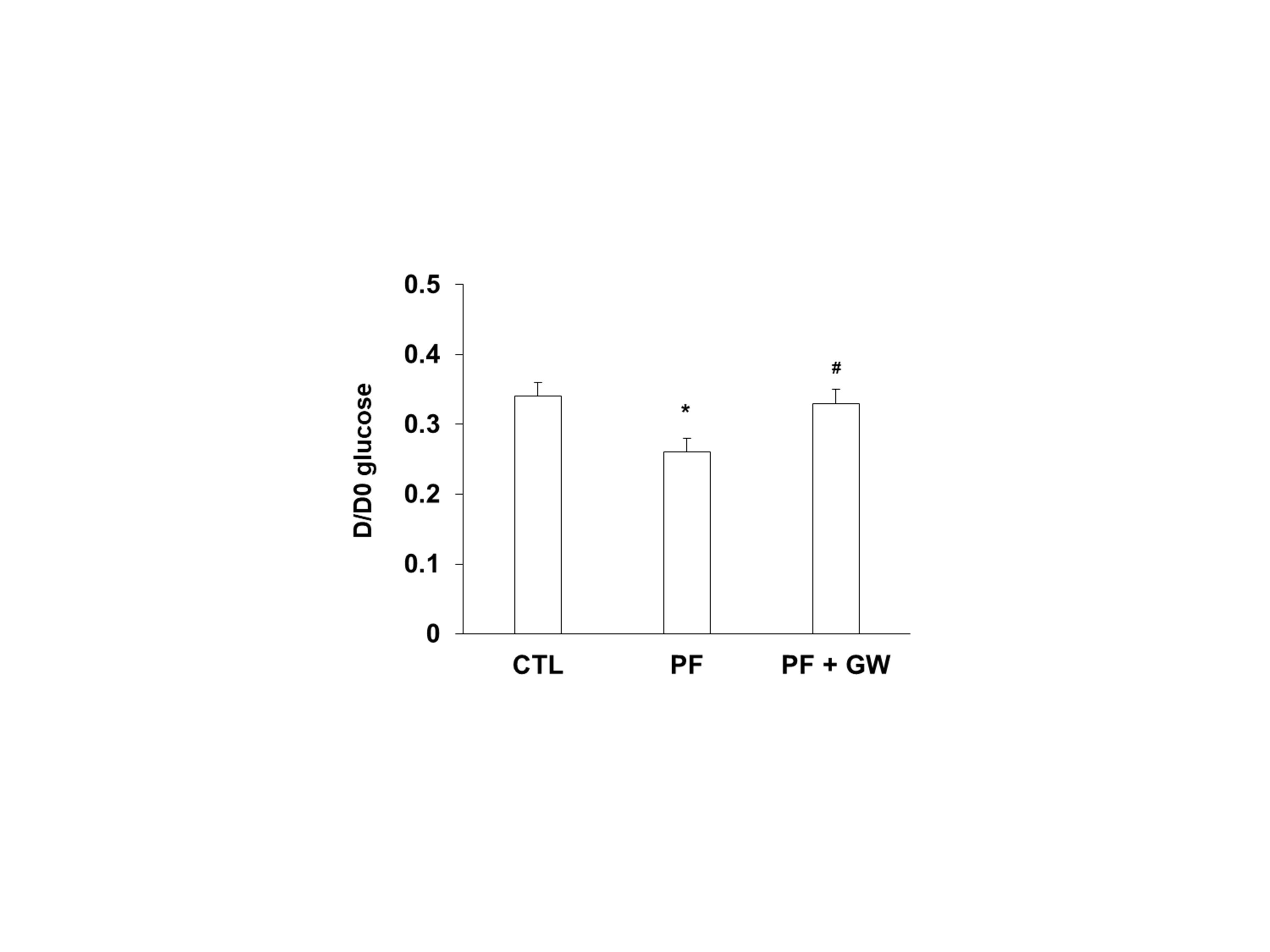

Supplement: Supplementary file 1 [file ijms-22-04739-s001.zip › Figure S3.jpg]

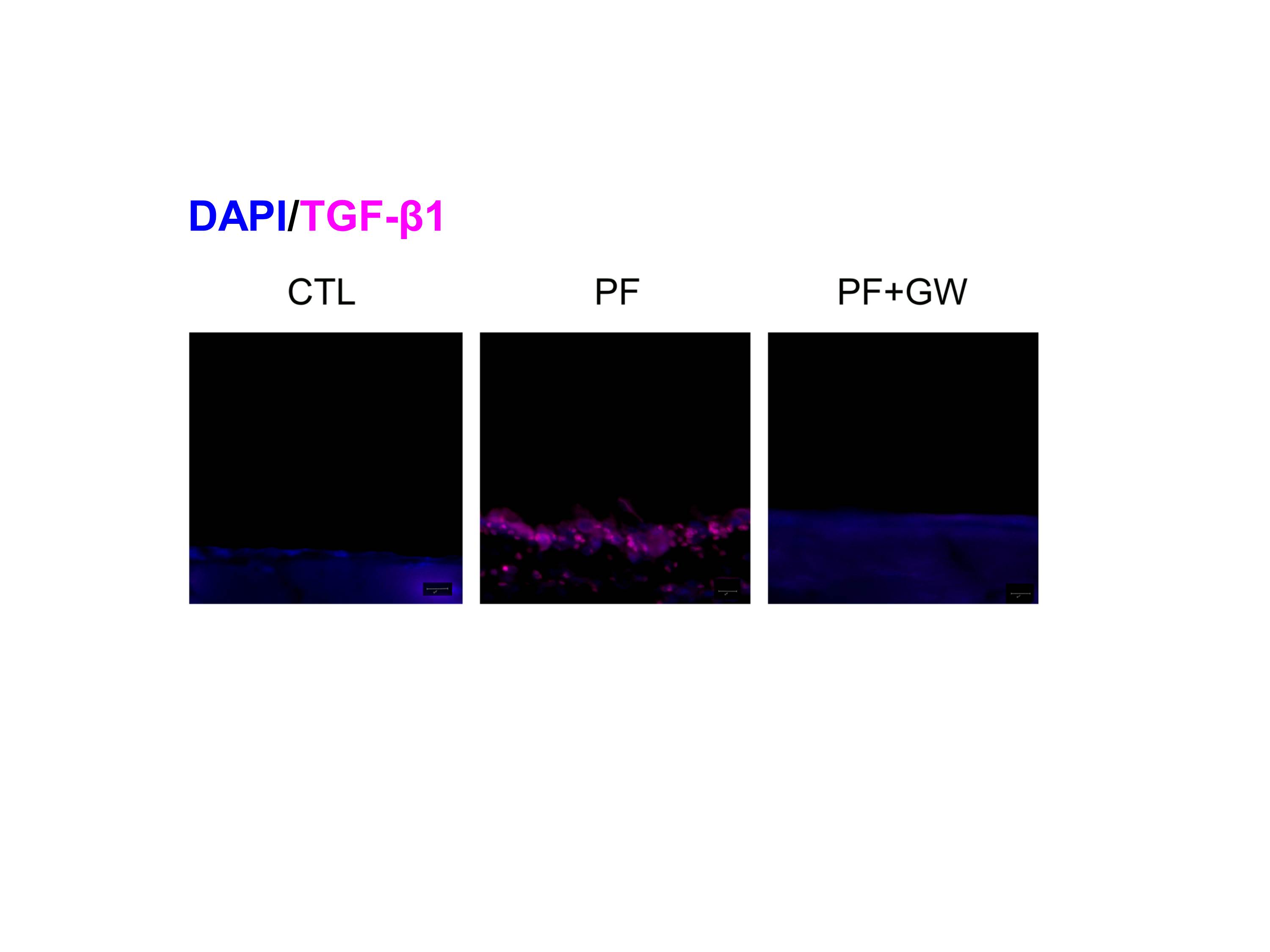

Supplement: Supplementary file 1 [file ijms-22-04739-s001.zip › Figure S4A.jpg]

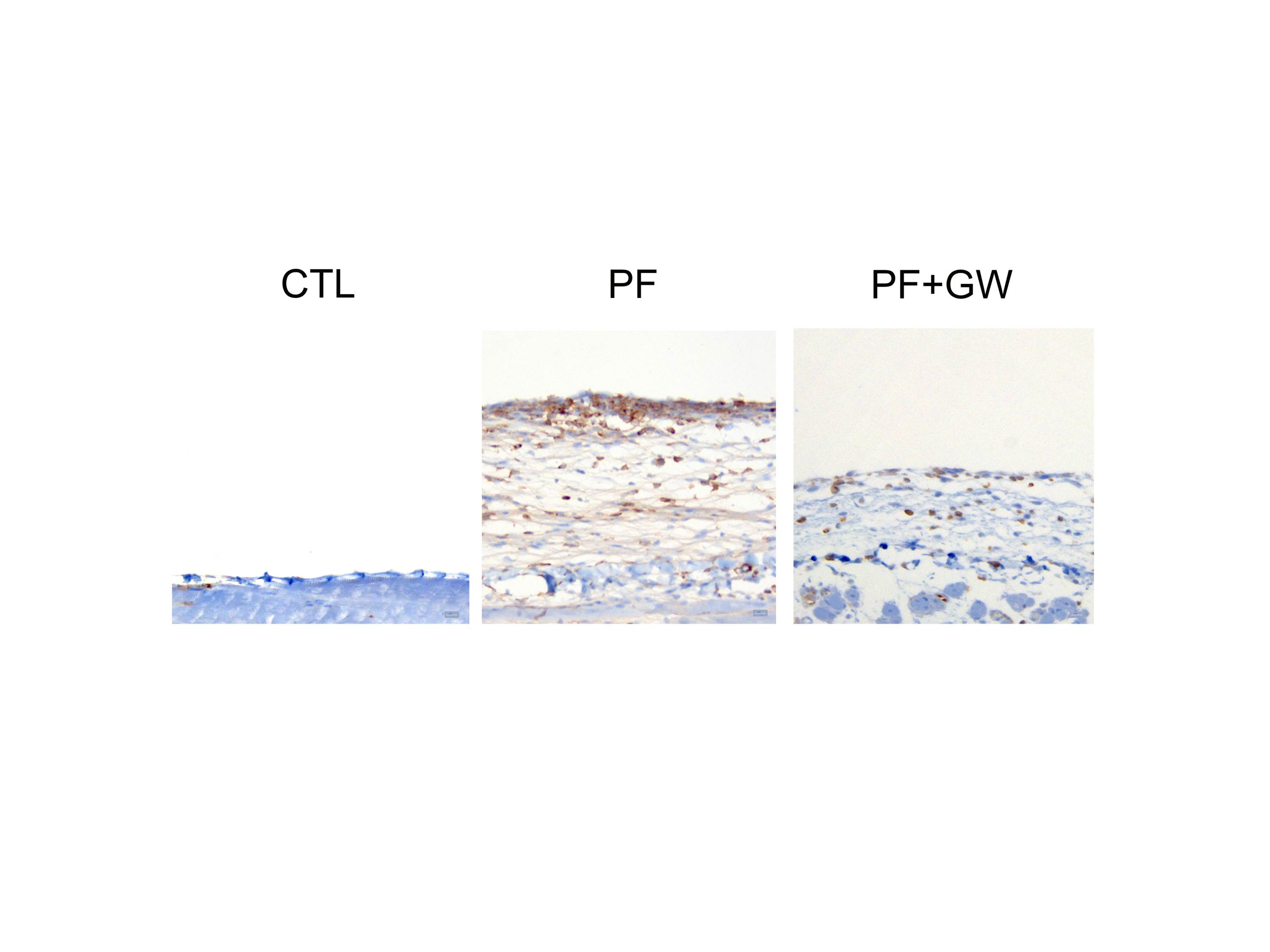

Supplement: Supplementary file 1 [file ijms-22-04739-s001.zip › Figure S4B.jpg]

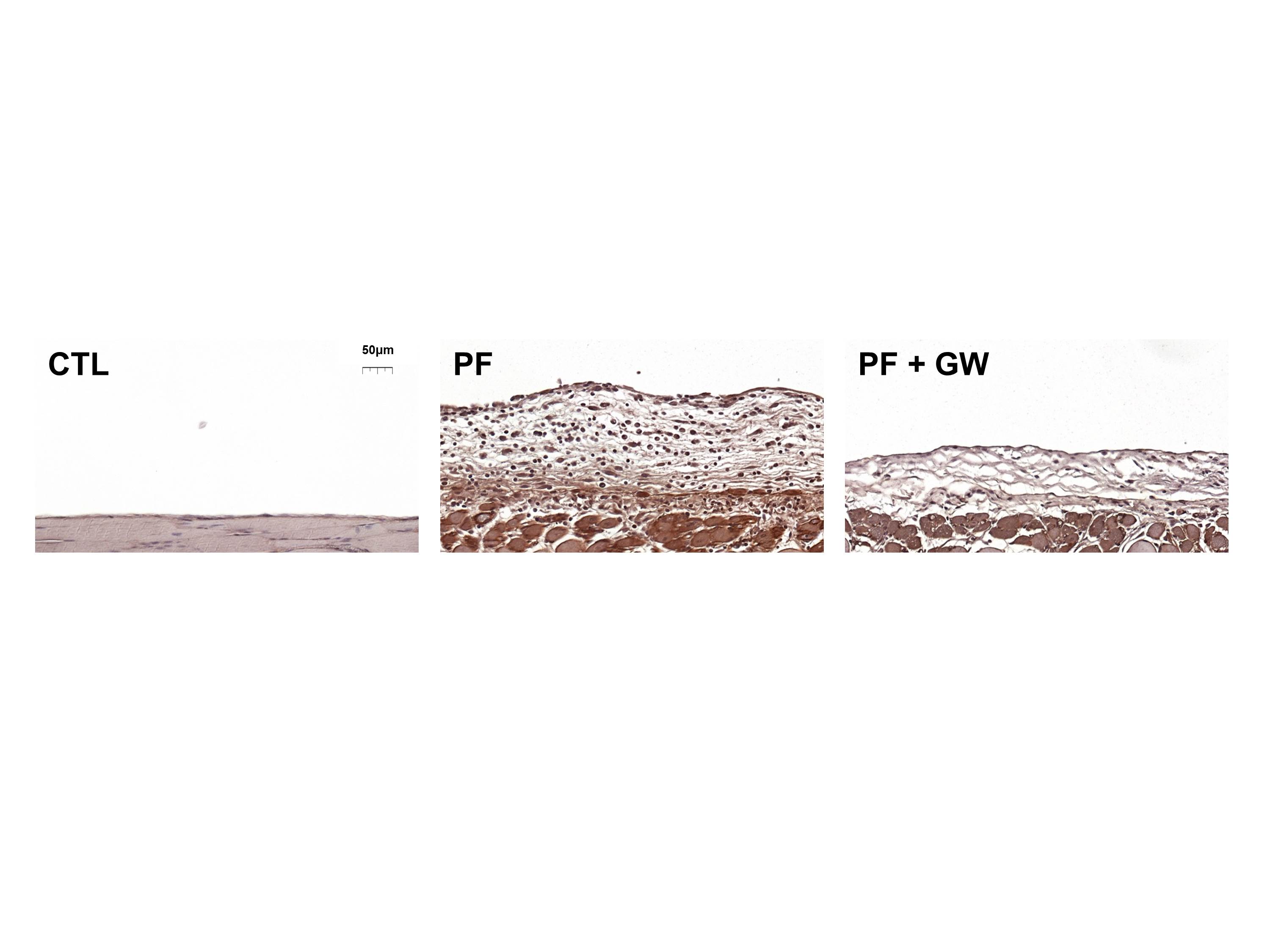

Supplement: Supplementary file 1 [file ijms-22-04739-s001.zip › Figure S5A.jpg]

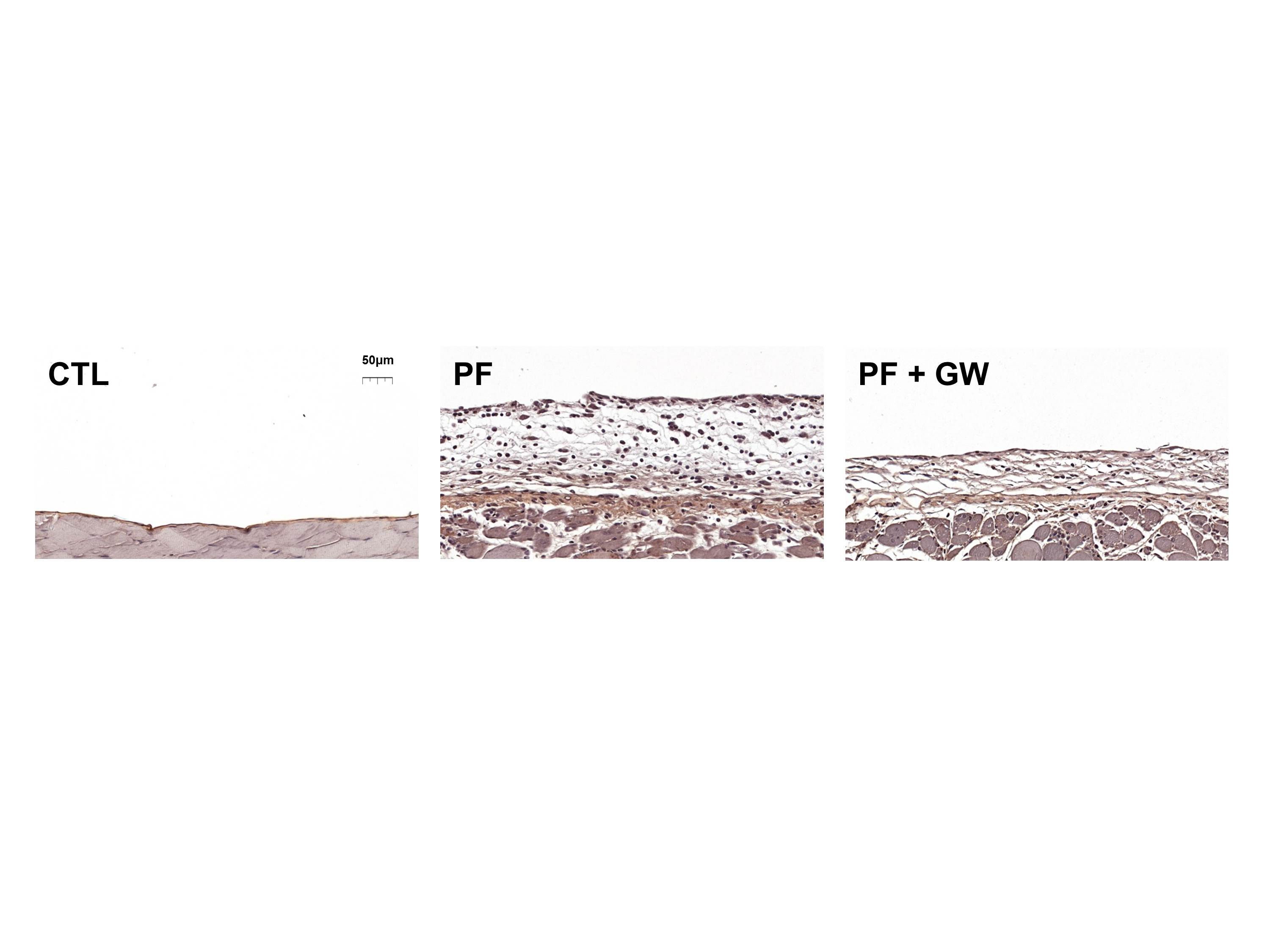

Supplement: Supplementary file 1 [file ijms-22-04739-s001.zip › Figure S5B.jpg]
